# The Role of Solidified Phases on the Hot Cracking of a Large-Size GH4742 Superalloy Ingot

**DOI:** 10.3390/ma17030614

**Published:** 2024-01-27

**Authors:** Liang Zhang, Lei Wang, Yang Liu, Xiu Song, Teng Yu, Ran Duan

**Affiliations:** 1 Key Laboratory for Anisotropy and Texture of Materials, Ministry of Education, School of Materials Science and Engineering, Northeastern University, Shenyang 110819, China; zhangliangdd@163.com (L.Z.); liuyang@mail.neu.edu.cn (Y.L.); songxiu@mail.neu.edu.cn (X.S.); 2 High Temperature Materials Division, Fushun Special Steel Co., Ltd., Fushun 113001, China; yuteng0318@163.com; 3 Gaona Aero Material Co., Ltd., Beijing 100081, China; duanran2019@163.com

**Keywords:** hot cracking, η phase, MC carbide, segregation degree, superalloy

## Abstract

The effect of solidified phases on the hot cracking behaviour of a large-size GH4742 superalloy ingot produced using vacuum induction melting (VIM) is investigated in order to improve the quality of the final product. The results show that the solidification order of the ingot is γ matrix, MC carbide, η phase and γ′ phase. Among them, the MC carbide and the η phase solidified in the mushy zone. The volume fraction of both the η phase and the MC carbide in the cracked zone is higher than that in the non-cracked zone, and a significant number of η phases are distributed near the hot cracks. The formation of solidified phases not only induces stress concentration at η phase/γ matrix interfaces but also reduces the ability of liquid feeding during solidification, thus promoting hot crack formation. It is believed that by controlling the segregation degree of both Nb and Ti, the volume fraction of η phases and MC carbides can be reduced to prevent hot cracking of the GH4742 superalloy VIM ingot.

## 1. Introduction

Nickel-based superalloys are widely used as hot parts in important industries, such as aerospace engines, land-based gas turbines and nuclear reactors, due to their superior strength, considerable creep resistance, good corrosion resistance and microstructural stability at elevated temperatures [1,2,3]. These excellent properties are attributed to high alloying, which typically contains more than five different alloying elements. GH4742, as one of the precipitation-strengthening nickel-based superalloys, is widely used in aerospace engines [4]. Alloying elements such as Co, Cr and Mo are solidly dissolved in the alloy for solution strengthening, while elements such as Nb, Ti and Al form the γ′ phase for precipitation strengthening [5,6]. On the other hand, the excessive addition of alloying elements can result in the formation of more complicated or undesirable solidified phases during solidification, which can easily lead to the formation of hot cracks.

Hot cracking easily occurs in the large-size ingots produced using vacuum induction melting (VIM). Meanwhile, the VIM ingot is used as the basic electrode used for the subsequent electroslag remelting (ESR) and vacuum arc remelting (VAR) processes in the modern melting process of superalloys. Nowadays, with the development of the advanced equipment manufacturing industry, there is an increasing demand for large-size and high-quality superalloy components. To meet this demand, it is necessary to produce much larger superalloy VIM ingots. However, solidification defects such as porosity and hot cracks are usually generated in the large-size superalloy VIM ingots. In practice, the tendency to hot crack increases with the size of the VIM ingot. The presence of cracks in VIM ingots leads to current or voltage instability in the subsequent remelting process (VAR or ESR), resulting in white spot or freckle defects that cannot be removed by heat treatment or hot working and are eventually inherited in the finished components. These metallurgical defects not only affect component reliability but also reduce production efficiency.

Hot cracking has received much attention from many researchers, and it has been found that hot cracking usually occurs in the mushy zone where the solid fraction is close to one [7]. Numerous metals, such as aluminium alloys [8,9], magnesium alloys [10,11] and stainless steels [12], are susceptible to hot cracking. Some researchers have found that hot cracking is a complicated phenomenon that can be influenced by mechanical and non-mechanical factors [13,14,15,16,17,18]. Li et al. investigated the effects of pouring temperature and mould temperature on the hot cracking tendency of Al-Cu alloys. The results showed that the hot cracking severity decreased with increasing mould temperature [19]. Davis et al. found that grain refinement can reduce the hot cracking tendency of the AZ91D alloy [20]. Du et al. investigated the role of the Ca/Al ratio on the hot cracking tendency of a Mg-Al-Ca alloy. The results show that a high Ca/Al ratio can reduce the tendency for hot cracking [21]. However, there is little research on the hot cracking behaviour of large-size superalloy VIM ingots.

Until now, previous investigations on the GH4742 superalloy have mainly focused on its microstructure and mechanical properties, while reports on the hot cracking behaviour of large-size VIM ingots are limited. Therefore, it is important to clarify the hot cracking mechanism of large-size GH4742 superalloy VIM ingots. In the present study, the effect of solidified phases on hot cracking behaviour is investigated to provide technical support for the production of crack-free superalloy VIM ingots.

## 2. Materials and Methods

**Figure 1:**
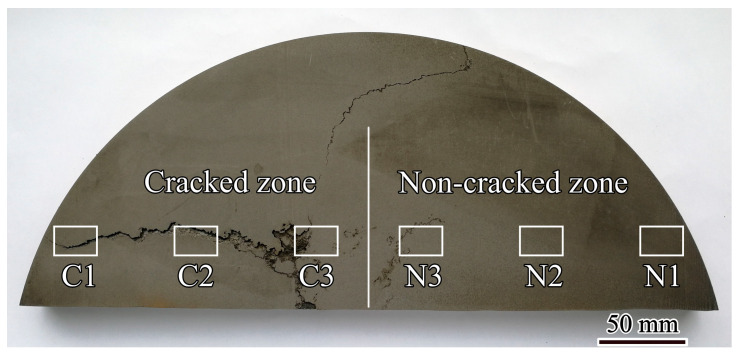
Photograph of the GH4742 superalloy VIM ingot sample [22].

The GH4742 superalloy ingot with a diameter of 430 mm and length of 2200 mm was produced using VIM with the following main chemical compositions (wt.%): 0.073 C, 14.40 Cr, 2.78 Al, 2.59 Ti, 2.42 Nb, 4.95 Mo, 10.40 Co and balance Ni. Figure 1 shows the transverse section of the semi-cylinder sample cut from the middle length of the VIM ingot. The sample had a cracked zone on the left and a non-cracked zone on the right. In the cracked zone, macro-cracks extended from the centre to the edge. The specimens cut from the edge, half radius and centre in the cracked zone are labelled C1, C2 and C3, respectively. The specimens cut from the edge, half radius and centre in the non-cracked zone are labelled N1, N2 and N3, respectively. Some microcracking can be observed near the N3 specimen.

Non-equilibrium solidification of GH4742 superalloy was calculated according to its chemical composition by using the nickel-based superalloy database in JMatPro software version 7.0. The morphological characteristics of the solidified phase, shrinkage porosity and hot cracking were examined using a scanning electron microscope (SEM, JSM-7001F, JEOL Ltd., Tokyo, Japan) equipped with an energy dispersive spectroscope (EDS). The specimens were ground and polished using the standard metallographic method and then electrolytically etched with a 15 g CrO_3_ + 10 mL H_2_SO_4_ + 150 mL H_3_PO_4_ solution at 3.5 V for about 20 s. An Olympus GX71 optical microscope (OM) was used to observe shrinkage porosity on as-polished but un-etched specimens. The volume fraction of solidified phases and shrinkage porosities were measured using ImageJ 1.41o software based on SEM and OM images, respectively. Transmission electron microscopy (TEM, JEM-2100F, JEOL Ltd., Tokyo, Japan) analysis was also carried out to identify the solidified phase. The TEM specimens were first mechanically ground to a thickness of about 50 μm and then punched into a round disc of 3 mm diameter. These discs were thinned by twin-jet electrolytic polishing with an electrolyte containing 10% HCOl_4_ + 90% C_2_H_5_OH at 13.5 V and −30 °C. The elemental distribution of solidified phases was measured using electron probe microanalysis (EPMA, JXA-8530F, JEOL Ltd., Tokyo, Japan).

The electron backscatter diffraction (EBSD) was performed using a Crossbeam550 SEM equipped with an Oxford instruments EBSD detector. The EBSD specimens were electrolytically polished in a solution of 100 mL H_2_SO_4_ + 300 mL CH_3_OH at 15 V for about 10 s. The phase map, inverse pole figure (IPF) map and kernel average misorientation (KAM) map were then analysed using Aztec 2.2 software.

## 3. Results

### 3.1. Solidification Behaviour

**Figure 2:**
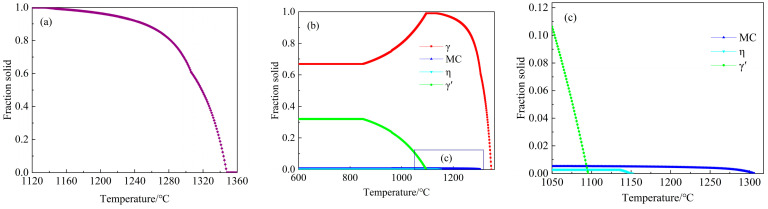
Thermodynamic calculation results of GH4742 superalloy: (**a**) solid fraction as a function of temperature; (**b**) non-equilibrium phase diagram; (**c**) enlarged part in (**b**).

The non-equilibrium solidification of the GH4742 superalloy, calculated using JMatPro software, is shown in Figure 2. The solidification started with the crystallisation of the γ matrix, and the corresponding liquidus temperature is 1347.82 °C. When the liquid fraction is zero and the corresponding solidus temperature is 1135.00 °C, MC carbides solidify at 1306.17 °C when the solid fraction is 0.61, indicating that MC carbide forms in the medium of the mushy zone. The η phases solidify at 1152.00 °C when the solid fraction is 0.99, indicating that the η phase forms at the end stage of solidification. On further cooling, γ′ phases precipitate from the supersaturated γ matrix at 1095.55 °C. Therefore, the solidification sequence of the GH4742 superalloy is liquid → γ phase → MC carbide → η phase → γ′ phase.

### 3.2. Characteristics of the Solidified Phases

**Figure 3:**
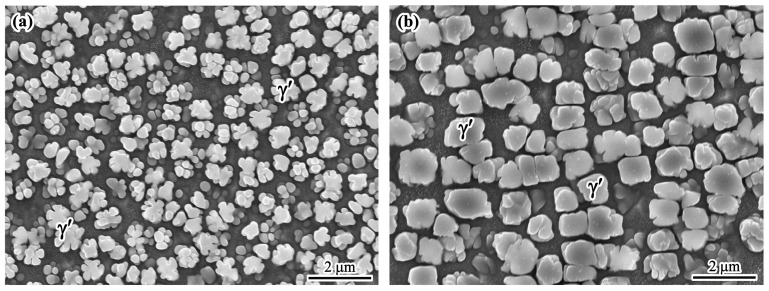
SEM images of the γ′ phase: (**a**) in the dendrite core; (**b**) in the interdendritic region.

Figure 3a,b shows the morphologies of the γ′ phase in the dendritic core and interdendritic region, respectively. It can be seen that the γ′ phase shows a petaloid morphology in the dendrite core and a cubic morphology in the interdendritic region. It can be noticed that the average size of the γ′ phase in the interdendritic region is larger than that in the dendrite core. The difference in morphologies may be related to the elemental fluctuation [23].

**Figure 4:**
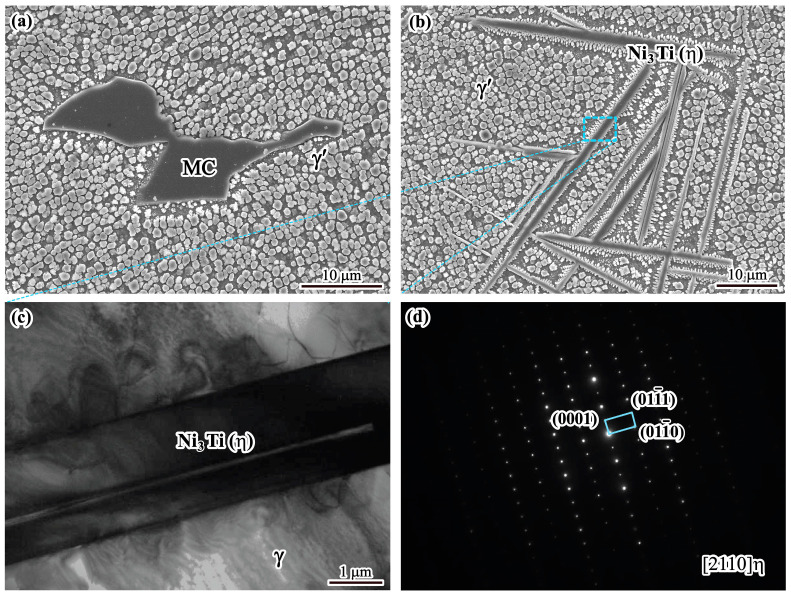
Microstructure of solidified phases: (**a**) SEM image of MC carbide; (**b**) SEM image of η phase; (**c**) TEM image of η phase; (**d**) selected area diffraction pattern.

In addition to the petaloid and cubic γ′ phases, the blocky phase (Figure 4a) and acicular phase (Figure 4b) are also found in the interdendritic region. The EDS analysis result shows that the blocky phase has the following composition (at.%): C: 59.79, Nb: 26.46, Ti: 11.99 and Ni: 1.76. It shows that the blocky phase is enriched in Nb, Ti and C, and it matches the stoichiometric ratio of MC. These phases have a large aspect ratio and are arranged close and parallel to each other, forming a colony-like configuration, as shown in Figure 4b. EDS analysis shows that these acicular phases are enriched with Ni, Ti and Al elements. TEM images and the corresponding selected area diffraction pattern as shown in Figure 4c,d indicate that the acicular phase is the η phase.

**Figure 5:**
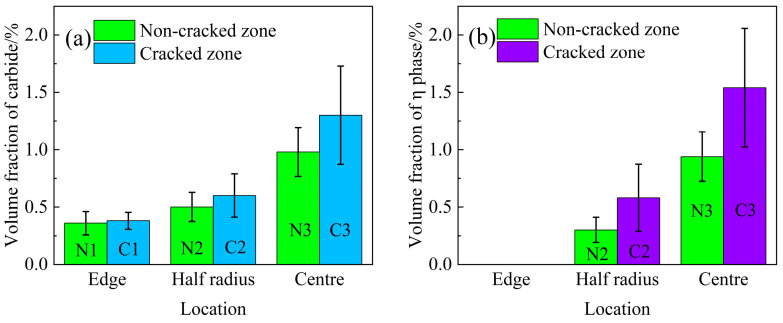
Volume fraction of the solidified phase in different locations of the ingot: (**a**) MC carbide; (**b**) η phase.

Figure 5a,b shows the statistical results of the volume fraction of MC carbide and η phase in different locations in the ingot, respectively. As shown in Figure 5a, the volume fraction of MC carbide increases from the edge to the centre of the ingot. There are no η phases at the edge of the ingot, as shown in Figure 5b, neither in the cracked zone nor in the non-cracked zone. The η phases appear at the half radius and the centre of the ingot, and the volume fraction is highest at the centre of the ingot. The formation of the η phase is strongly influenced by the cooling rate. The faster cooling rate is able to suppress the precipitation of the η phase [24]. In fact, the precipitation of the η phase can be inhibited when the cooling rate reaches a certain level because the η phase-forming elements of both Nb and Ti are insufficient in the interdendritic region. During solidification, the cooling rate is highest at the edge of the ingot, so the precipitation of η phases is completely suppressed. The centre of the ingot has the lowest cooling rate, which causes more Nb and Ti to segregate into the residual liquid, promoting the formation of η phases. In addition, as shown in Figure 5, the volume fraction of MC carbides and η phases is higher in the cracked zone than that in the non-cracked zone because the segregation degree of Ti and Nb is higher in the cracked zone than that in the non-cracked zone, as can be seen in our previous work [22].

### 3.3. Shrinkage Porosity and Hot Cracking

**Figure 6:**
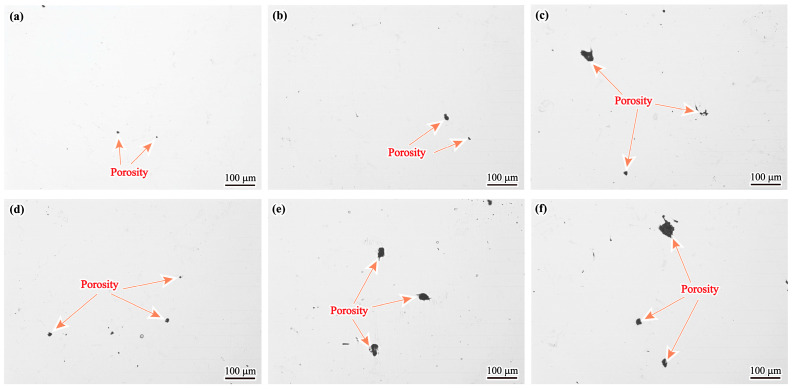
Microstructure of porosity in different specimens of the ingot: (**a**) N1; (**b**) N2; (**c**) N3; (**d**) C1; (**e**) C2; (**f**) C3.

Figure 6 shows the appearance and size of porosity in different specimens of the GH4742 superalloy VIM ingot. Compared with the porosity in the edge, those in the centre have larger sizes and anomalous morphologies. It indicates that the size of shrinkage porosity increases with decreasing cooling rate, which has been reported by Han et al. in IN738LC [25].

**Figure 7:**
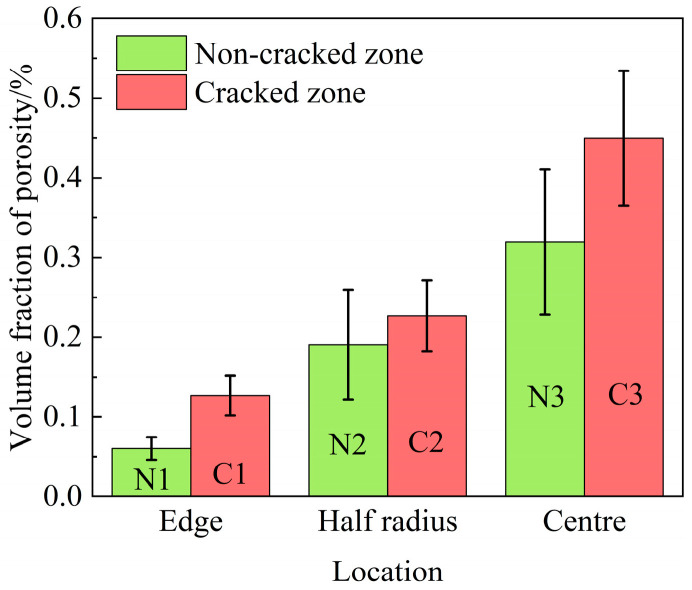
Volume fraction of porosity in different locations of the ingot.

Figure 7 shows the volume fraction of porosity in the different locations of the ingot. It shows that the volume fraction of porosity gradually increases from the edge to the centre of the ingot, and it is about 35% higher in the cracked zone than that in the non-cracked zone (at the centre).

**Figure 8:**
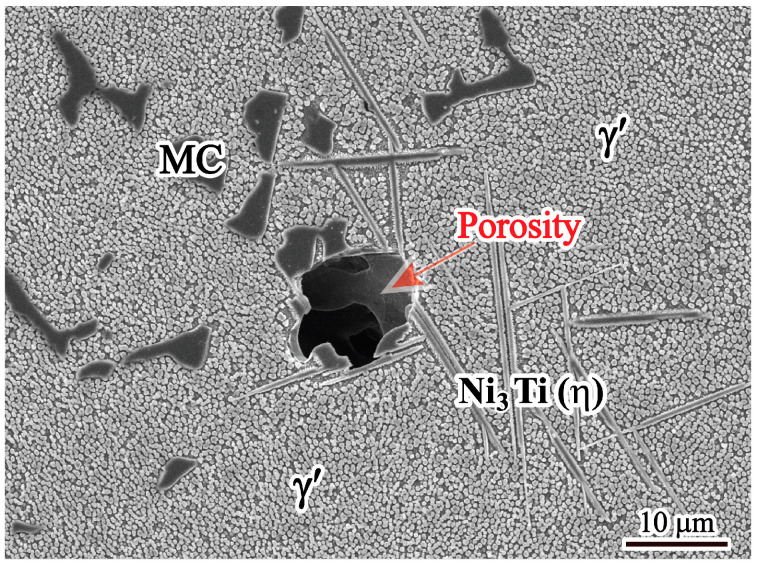
Microstructure of shrinkage porosity in the ingot.

The SEM morphology of the porosity is shown in Figure 8. The porosity has an irregular shape. It is well known that porosity is formed during solidification by shrinkage or gas evolution. Therefore, the porosity types can be divided into shrinkage porosity and gas porosity, which are determined using morphological characteristics. Gas porosity usually has a spherical shape, while shrinkage porosity has an irregular shape [26]. Based on the SEM image shown in Figure 8, the porosity in the present study has an irregular shape, indicating that it is shrinkage porosity. It is also because the vacuum level is very high during VIM and the raw materials are dry with low gas content that gas porosity can be suppressed [27]. In addition, there are MC carbides and a large amount of η phases around the shrinkage porosity.

**Figure 9:**
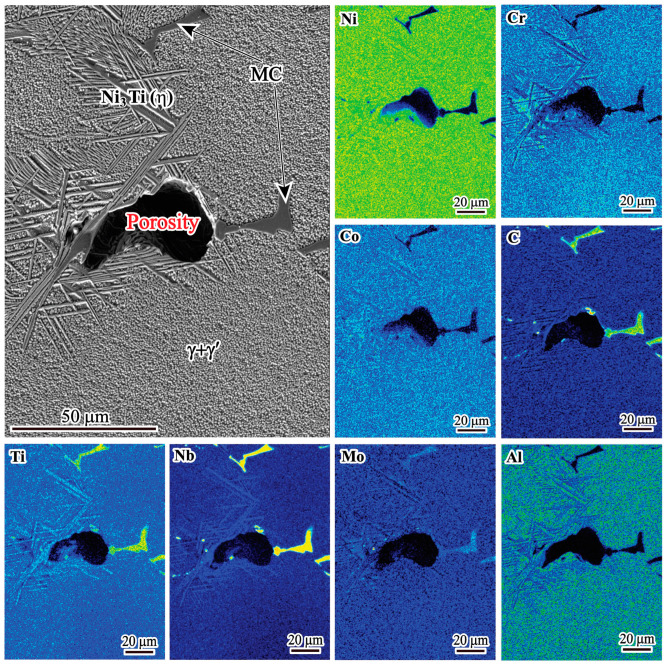
EPMA mappings of MC carbide and η phase near the shrinkage porosity in the ingot.

To investigate the reason for the coexistence of MC carbides and η phases, EPMA measurements were performed in the region containing the MC-η network and shrinkage porosity, and the results are shown in Figure 9. It is obvious that both Nb and Ti are heavily enriched in MC carbides. Meanwhile, a high concentration of Ti is found in the acicular η phase. As solidification proceeds, both Nb and Ti elements are continuously segregated into the residual liquid, providing favorable conditions for the formation of MC carbides and η phases. Based on JMatPro calculation results, the MC carbide solidified earlier than the η phase. Once the MC carbide solidifies, it indicates that a high concentration of Nb and Ti elements segregates in this region. As Nb preferentially concentrates in the MC carbide, Ti continuously segregates to the residual liquid, promoting the η phase solidified environment with MC carbide. As a result, MC carbide and η phase usually coexist in the interdendritic region, forming an MC-η network that impedes residual liquid feeding, leading to the formation of shrinkage porosity.

**Figure 10:**
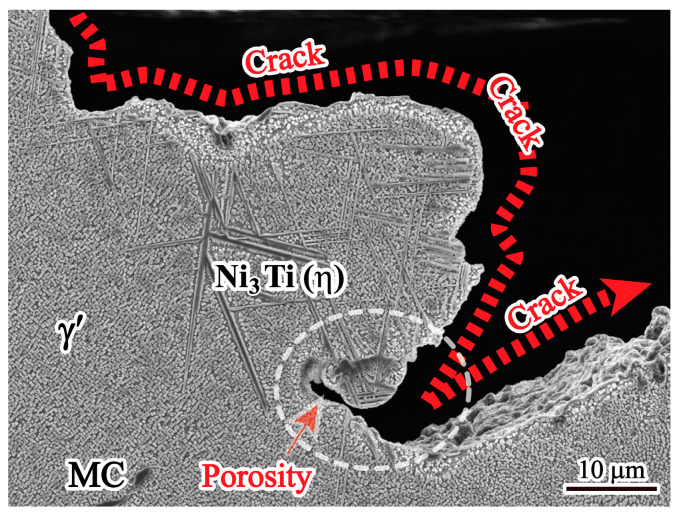
Microstructure of hot cracks in the ingot.

To investigate the origin of hot cracking, the microstructure near the cracks was also examined with SEM, and the result is shown in Figure 10. These cracks show relatively irregular and zigzag morphology, which is consistent with the characteristics of hot cracking. It is worth noting that η phases appear at the edges of these cracks.

**Figure 11:**
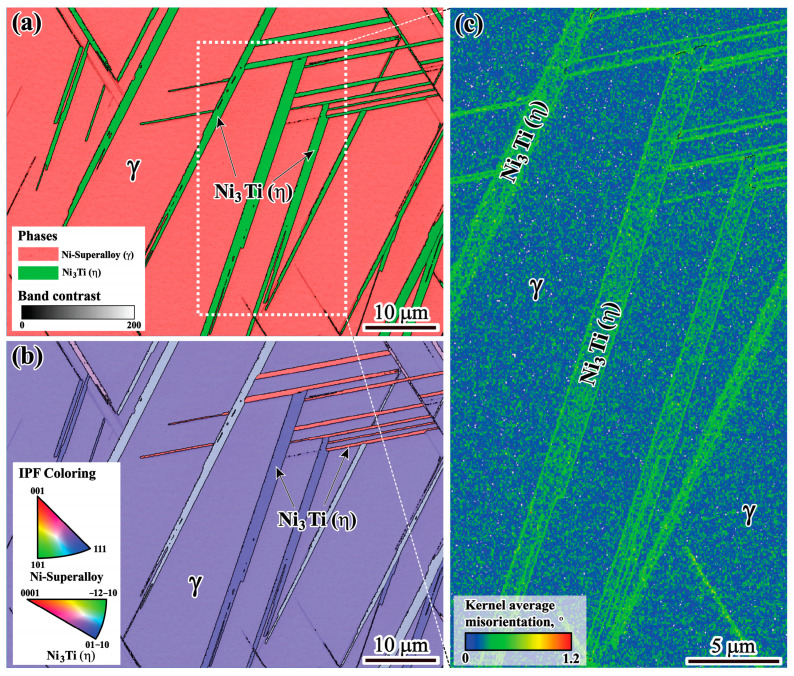
EBSD analysis of the ingot: (**a**) phase map; (**b**) IPF map; (**c**) KAM map.

Furthermore, the strain concentration around η phases is determined using KAM analysis. As shown in Figure 11, it is evident that a higher KAM density is concentrated around η phases, indicating that strain is concentrated around η phases.

## 4. Discussion

Based on the above results, the solidified phases of the large-size GH4742 superalloy VIM ingot include the MC carbide, the η phase and the γ′ phase, in addition to the γ matrix. In the mushy zone, hot cracking occurs with shrinkage porosity surrounding MC carbides and η phases. Therefore, it can be suggested that the formation of shrinkage porosity and hot cracking is closely associated with the formation of η phases and MC carbides.

### 4.1. Shrinkage Porosity Formation Mechanism

It is well known that the formation of shrinkage porosity is caused by both solidification shrinkage and a lack of liquid feeding in the final stage of solidification [28]. From Figure 8, it can be seen that shrinkage porosity usually occurs near the MC carbide and the η phase. The MC carbide exhibits a large blocky morphology, and the η phase tends to cluster together interactively. In addition, η phases usually form around MC carbide and touch each other to form an MC-η network in the interdendritic region. Such an MC-η network easily divides the residual liquid into isolated melt pools, which can block the liquid flow freely; thus, the residual liquid cannot be fed, resulting in the formation of shrinkage porosities. As shown in Figure 7, the volume fraction of shrinkage porosity has a direct correlation with the volume fraction of MC carbides and η phases (Figure 5a,b). This means that the higher the volume fraction of MC carbides and η phases, the higher the probability of shrinkage porosity formation. Therefore, the formation of shrinkage porosity is closely related to the MC carbides and η phases.

### 4.2. Hot Cracking Behaviour

Usually, hot cracking is mainly caused by insufficient liquid supply to the mushy zone after stress/strain aggravation [29,30]. Based on this point, the effect of the solidified phase on hot cracks can be divided into mechanical and non-mechanical aspects.

Mechanically, there are stresses induced by solidification shrinkage and thermal contraction because the VIM ingot cannot freely contract in the mould during solidification. In addition, there is high thermal stress during solidification due to non-uniform cooling rates and inhomogeneous thermal gradients throughout the large-size VIM ingot, which provides the driving force for hot crack formation. As a kind of nickel-based superalloy, the GH4742 superalloy has a high thermal expansion coefficient [31], which causes large volume shrinkage, resulting in a high level of stress during solidification. It is well known that hot cracking depends on the competition between stress and the inherent resistance of the semi-solid material to hot cracking [32].

Due to non-equilibrium solidification, a large amount of solidified phase is formed in the interdendritic region. As shown in Figure 5, the volume fraction of MC carbide and η phase in the cracked zone is higher than that in the non-cracked zone, indicating that the hot cracking tendency is proportional to the volume fraction of solidified phases. That can be attributed to the following two reasons: On the one hand, the formation of the MC carbide and the η phase in the interdendritic region consumed the strengthening elements Nb and Ti in the surrounding γ matrix, resulting in a decrease in strength around the solidified phases. On the other hand, both MC carbides and η phases are brittle compared with the γ matrix, which is difficult to adapt to solidification shrinkage. These solidified phases form in the interdendritic region, reducing the interdendritic bonding force between dendrites and the ductility of the alloy. 

Moreover, η phases tend to form at crack edges, indicating that the η phase region is more susceptible to hot cracking, as shown in Figure 10. It can be clearly seen from Figure 11 that a higher level of strain accumulation, determined using KAM density analysis, forms near the η phase/matrix interfaces, which is related to the strain incompatibility between the η phase and matrix caused by the inhomogeneous deformation ability of these two phases. The solidified phases form in the interdendritic region, which can destroy the homogeneity of the alloy and cause stress concentration. The brittle solidified phases of both MC carbides and η phases will act as a nucleation source for hot cracking under stress. Therefore, the η phase plays a predominant role in the formation of hot cracks during solidification. 

In addition, hot cracking is also related to shrinkage porosity. It can be seen from Figure 7 that the volume fraction of shrinkage porosity in the cracked zone is higher than that in the non-crack zone, indicating that the higher the shrinkage porosity, the greater the hot cracking tendency. This is because the shrinkage porosities are easily interconnected under stress. The higher the shrinkage porosities, the more easily they can coalesce with each other. In other words, in the region of shrinkage porosity, stress concentration occurs, and the effective area to support deformation during solidification is reduced, thus promoting the formation of hot cracking. Therefore, shrinkage porosities are also considered the nucleation site of hot cracking [33].

Among the non-mechanical aspects, the liquid feeding ability in the final stage of solidification can be considered another important factor for hot cracking. Hot cracking easily occurs when there is insufficient liquid feeding to compensate for solidification shrinkage [34,35,36]. During solidification, the dendrite size increases as the melt temperature decreases, and the liquid feed channel gradually narrows. Due to element segregation, Nb and Ti elements are enriched in the interdendritic region, which promotes a large number of MC carbide and η phase solidifications in the interdendritic region. When solidified phases are formed in the residual liquid, a coexistence state of liquid and solidified phase is formed. Because of that, the residual liquid with the solidified phase has a higher viscosity than the residual liquid without the solidified phase. At the same time, the increase in viscosity of the residual liquid between dendrites can slow down the flow of the residual liquid. 

In addition, once the MC carbides and η phases are formed in the interdendritic region, they bridge with each other to form an MC-η network. Liquid flow between dendrites is blocked by the formation of the MC carbide and the η phase. The more the MC carbides and the η phases in the residual liquid, the weaker the ability of liquid feeding. Therefore, the MC carbide and the η phase formed in the interdendritic region reduce liquid feeding and promote hot cracking. 

**Figure 12:**
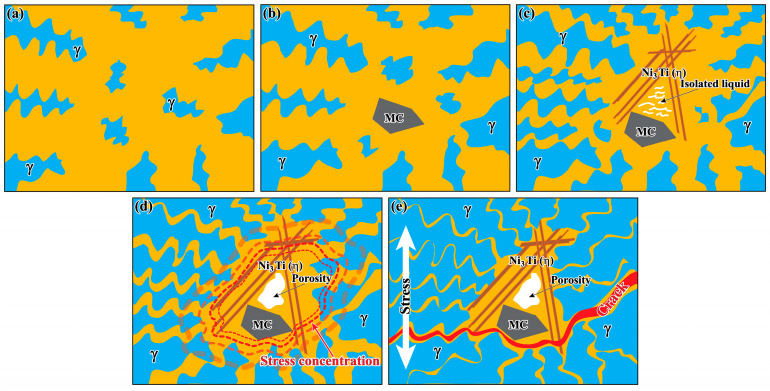
The schematic diagram of hot cracking behaviour in the GH4742 superalloy VIM ingot. (**a**) γ dendrites formation; (**b**) MC carbide formation; (**c**) η phases form near the MC carbide; (**d**) porosity formation; (**e**) hot cracking formation.

Based on the above, the schematic diagram of the hot cracking behaviour of the GH4742 superalloy VIM ingot can be summarised as shown in Figure 12. At the initial solidification stage, γ dendrites grow first, no thermal strain occurs and residual liquid flows freely between dendrites (Figure 12a). As solidification proceeds, both the size and volume fraction of γ dendrite increase, and MC carbide forms in the interdendritic region (Figure 12b). Subsequently, η phases form near the MC carbide, forming an MC-η network. And the residual liquid in the MC-η network becomes an isolated melt pool (Figure 12c). As the temperature decreases, the isolated liquid shrinks, and the free passage of the residual liquid is blocked with the MC-η network, forming a shrinkage porosity at the position of the isolated molten pool. With further cooling, a stress concentration zone is formed around the MC carbide, the η phases and the shrinkage porosity region (Figure 12d). At the end of solidification, the liquid feed channel is blocked by the MC-η network, and the shrinkage porosity can act as the initiation of hot cracking under stress (Figure 12e). In short, hot cracking easily initiates around the shrinkage porosities and propagates along the MC carbides and η phases, which is the stress concentration region.

## 5. Conclusions

1. The phase solidification sequence of the GH4742 superalloy ingot is the γ phase, the MC carbide, the η phase and the γ′ phase. The MC carbides and the η phases are mainly formed in the mushy zone, which hinders liquid feeding and induces shrinkage porosity.
2. The volume fraction of η phases, MC carbides and shrinkage porosities is higher in the cracked zone than that in the non-cracked zone. The hot cracking tendency increases with the formation of η phases and MC carbides.
3. During the cooling process, the stress concentration occurs in the η phases and the MC carbides within the shrinkage porosity zone, which promotes the hot crack formation of the GH4742 superalloy ingot.
4. In order to prevent hot cracking, it is suggested to reduce the number of η phases and MC carbides, thereby reducing the stress concentration. Controlling the segregation degree of both Nb and Ti is considered the most important to improve the casting quality of the GH4742 superalloy ingot.

## Data Availability

Data are contained within the article.
